# Enhancing Photovoltaic Performance Using Broadband Luminescent Down-Shifting by Combining Multiple Species of Eu-Doped Silicate Phosphors

**DOI:** 10.3390/nano7100340

**Published:** 2017-10-21

**Authors:** Wen-Jeng Ho, Yu-Tang Shen, Jheng-Jie Liu, Bang-Jin You, Chun-Hung Ho

**Affiliations:** 1 Department of Electro-Optical Engineering, National Taipei University of Technology, Taipei 10608, Taiwan; t103658029@ntut.edu.tw (Y.-T.S.); jjliu@mail.ntut.edu.tw (J.-J.L.); t105658002@ntut.edu.tw (B.-J.Y.); 2 Realtek Semiconductor Corporation, No. 2, Innovation Road II, Hsinchu Science Park, Hsinchu 30076, Taiwan; cunhonho@gmail.com

**Keywords:** Eu-doped silicate phosphors, luminescent downshifting (LDS), spin-on film technique, silicon solar cell

## Abstract

This paper demonstrates the application of a broadband luminescent downshifting (LDS) layer with multiple species of europium (Eu)-doped silicate phosphors using spin-on film technique to enhance the photovoltaic efficiency of crystalline silicon solar cells. The surface morphology of the deposited layer was examined using a scanning electron microscope (SEM). The chemical composition of the Eu-doped silicate phosphors was analyzed using energy-dispersive X-ray spectroscopy (EDS). The fluorescence emission of the Eu-doped silicate phosphors was characterized using photoluminescence (PL) measurements at room temperature. We also compared the optical reflectance and external quantum efficiency (EQE) response of cells with combinations of various Eu-doped phosphors species. The cell coated with two species of Eu-doped phosphors achieved a conversion efficiency enhancement (∆*η*) of 19.39%, far exceeding the ∆*η* = 15.08% of the cell with one species of Eu-doped phosphors and the ∆*η* = 8.51% of the reference cell with the same silicate layer without Eu-doped phosphors.

## 1. Introduction

The conversion efficiency of single band-gap solar cells is constrained by the need to match the band-gap of the cell material to the radiation spectrum of the sun. The Shockley–Queisser limit of 31% for a single junction semiconducting solar cell can be reached for materials with a band-gap of 1.1–1.3 eV [1]. The band-gap of crystalline silicon is 1.1 V; however, the conversion efficiency of crystalline silicon solar cells at Ultraviolet-blue (UV–blue) wavelengths remains relatively low due to high surface recombination loss and low responsivity within the UV–blue wavelength band [2]. Anti-reflective coatings and textured or structured surfaces are commonly used to reduce surface reflection, surface recombination, and improve light trapping in order to enhance conversion efficiency [3,4]. A number of approaches have been devised to enhance conversion efficiency at short wavelengths, including the down-conversion (DC) or luminescent down-shifting (LDS) of the incident spectrum [5,6,7,8,9,10,11,12,13]. The DC process involves conversion of high-energy incident photons into two lower energy photons. Trupke et al. reported that DC material distinct from the solar cell can be used to split photons with energy at twice the band-gap energy into two lower energy photons matching the band-gap of the solar cell [14]. DC is meant to increase the current of the solar cell by increasing the proportion of photons it absorbs, while retaining its voltage characteristics to increase the overall efficiency of the system. LDS is similar to DC; however, only one photon is emitted [15]. LDS is a passive method involving the application of a luminescent species above the solar cell to absorb light of short wavelengths and re-emit it at longer wavelengths. This requires materials with a high photoluminescent quantum yield (PLQY) and large Stokes shift LDS to improve the efficiency of crystalline silicon solar cells [16,17]. The high luminescence quantum efficiency and large Stokes-shift europium-ion (Eu^+3^) complexes make them excellent LDS species for crystalline silicon solar cells [18,19,20,21,22,23,24,25]. Despite extensive research in this area, little effort has gone into extending the LDS band by combining multiple species of Eu-doped phosphors [26,27].

In this study, we investigated crystalline silicon solar cells coated with a layer comprising one or two species of 3 wt % europium-doped (Eu-doped) silicate phosphors. The coatings were applied using spin-on film technique. We examined the surface coverage and dimensions of the phosphor particles using scanning electron microscopy (SEM) in conjunction with Image-J software (National Institute of Mental Health, Bethesda, MD, USA). The chemical composition of the Eu-doped phosphors was analyzed using energy-dispersive X-ray spectroscopy (EDS). The optical properties of the Eu-doped phosphors layer were examined by photoluminescence (PL) and UV–vis–NIR (Near-infrared) spectrophotometer at room temperature. Measurements of PL, optical reflectance, and external quantum efficiency (EQE) response were used to examine the effectiveness of LDS based on Eu-doped phosphors. Finally, we used photovoltaic current density-voltage (J–V) measurements to compare the photovoltaic performance of crystalline solar cells with two species of Eu-doped phosphors, one species of phosphors, or a no phosphors layer.

## 2. Experiments

### 2.1. Preparation and Characterization of Eu-Doped Silicate Phosphors Layer

A SiO_2_ coating mixed with one species of 3 wt % Eu-doped silicate phosphors was first applied to clean silicon substrates via spin-on coating. The coating solution comprised 1.94 g of Silicafilm-5000 (Emulsitone Company product, Whippany, NJ, USA.) combined with 0.06 g of Eu-doped silicate phosphors powder (InteMatix Company product, Fremont, CA, USA). The species of Eu-doped silicate phosphors powder included G2060TM (emission wavelength of 512 nm), EY4156 (emission wavelength of 550 nm), and O6040 (emission wavelength of 610 nm). In other samples, a SiO_2_ coating was mixed with two species of 3 wt % Eu-doped silicate phosphor. This was achieved by combining 1.94 g of Silicafilm-5000 with 0.03 g of the first silicate phosphor species and 0.03 g of the second silicate phosphor species; i.e., combining G2060TM + O6040 species, EY4156 + O6040 species, or G2060TM + O6040 species. The corresponding emission wavelengths of the phosphors would therefore be 512 nm + 550 nm, 550 nm + 610 nm, or 512 nm + 610 nm, respectively. The solutions were spin-coated on clean silicon substrates at 3000 rpm for 60 s before being baked at 200 °C for 30 min under an air atmosphere. The solution was held on the samples for 15 s prior to spinning to increase coverage. As a control, we also produced samples with a 250-nm thick layer of pure silicate (SiO_2_) using the same Silicafilm-5000 without phosphor particles. We used the same coating parameters (i.e., spin speed of 3000 rpm for 60 s and baking at 200 °C for 30 min) for all samples. The surface morphology and chemical composition of the samples with Eu-doped silicate phosphors layer were examined using scanning electron microscopy (SEM, Hitachi S-4700, Hitachi High-Tech Fielding Corporation, Tokyo, Japan) and energy-dispersive X-ray spectroscopy (EDS) (JSM-6500F, JEOL Ltd., Tokyo, Japan). The fluorescence emission of the Eu-doped silicate phosphors layers was confirmed by photoluminescence (PL; Ramboss 500i Micro-PL Spectroscopy, DONGWOO Optron, Korea) measurements at room temperature. The reflectance, LDS, and light scattering effects of the Eu-doped silicate layer with and without phosphors particles was characterized using an UV–vis–NIR spectrophotometer (PerkinElmer LAMBDA 35, Waltham, MA, USA).

### 2.2. Fabrication and Characterization of Silicon Solar Cells Coated with Eu-Doped Silicate Phosphors

**Figure 1:**
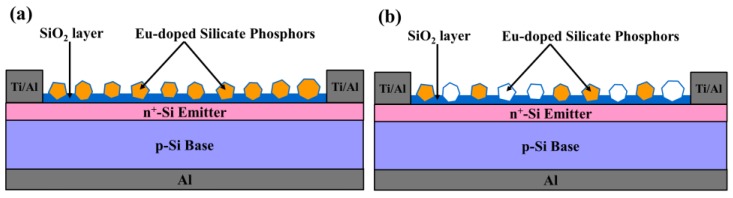
Schematic diagram showing silicon solar cells coated with SiO_2_ layer mixed with (**a**) one species and (**b**) two species of Eu-doped phosphors.

Figure 1 presents a schematic diagram showing silicon solar cells coated with a layer of silicate that included (a) one species or (b) two species of Eu-doped silicate phosphors. Bare silicon solar cells were first fabricated using a 525-μm-thick p-type crystalline silicon wafer (Czochralski (CZ): (100), 5 Ω·cm). After standard cleaning processes, an n^+^-Si emitter layer was applied via spin-on film processing using a liquid phosphorous source, followed by heat treatment in rapid thermal annealing (RTA) chamber at 900 °C for 2 min under ambient N_2_. Following the formation of the diffuse n^+^-Si layer, the phosphorous oxide remaining on the sample surface was removed using a buffered oxide etchant. Four-point probe resistivity and electrochemical capacitance-voltage (ECV) profiling revealed that the n^+^-Si emitter layer had a sheet resistance of 75 Ω/sq and a thickness of 350 nm. The peak in the phosphorus concentration at the surface was on the order of 10^20^ cm^−3^. E-beam evaporation and lift-off processing were used to deposit an Al film (300 nm in thickness) on the back-side as an electrode as well as an Al/Ti film (20 nm/300 nm) on the front-side as finger-electrodes. Finally, bare-type solar cells were obtained after annealing in RTA chamber at 450 °C for 10 min under ambient N_2_.

Improvement in the efficiency of the Eu-doped silicate phosphors layer was characterized by coating the bare solar cells with Silicafilm solution mixed with 3 wt % of phosphors powder. In this study, cells with layers containing one species or two species of Eu-doped silicate phosphor were used to identify the emission band of LDS effects on the photovoltaic performance of silicon solar cells. Photovoltaic J–V measurements under one-sun air mass (AM) 1.5G illumination as well as the external quantum efficiency (EQE) response from 300 nm to 1200 nm wavelengths were used to confirm the enhanced contribution from the Eu-doped silicate phosphor layers.

## 3. Results and Discussion

**Figure 2:**
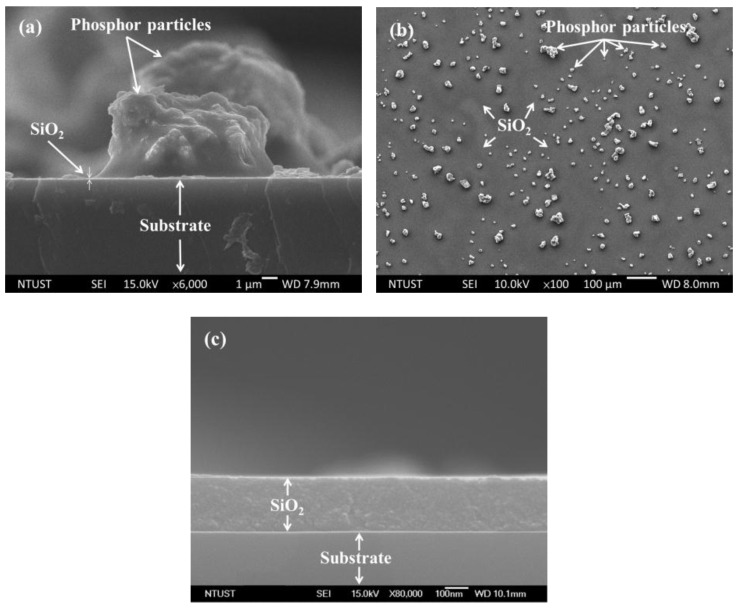
Scanning electron microscopy (SEM) images of silicon substrates with SiO_2_ layer mixed with phosphor particles: (**a**) cross-section; (**b**) top-view showing distribution of particles across surface; and (**c**) cross-section SEM image showing sample with a layer of SiO_2_ coated with the same Silicafilm-5000 without phosphor particles.

Figure 2 presents (a) cross-section and (b) top-view SEM images of Si-substrates spin-coated with a SiO_2_ layer mixed with Eu-doped silicate phosphors. The Eu-doped phosphor particles were approximately 5–20 μm in diameter. Some of the phosphor particles were not distributed uniformly across the surface. As shown in Figure 2b, some areas were shaded by the dense aggregation of particles which reflected the incident light, thereby lowering the conversion efficiency of the solar cells. Figure 2c presents a cross-section SEM image showing a sample with a layer of SiO_2_ coated with the same Silicafilm-5000 without phosphor particles. The thickness of the spin-coated SiO_2_ layer was approximately 240 nm. Top-view SEM images were analyzed using J-image software to compare the coverage of samples with one species or two species of Eu-doped silicate phosphors. Table 1 lists the coverage of samples with one-species or two-species of Eu-doped silicate phosphors. The coverage was similar across all samples.

**Figure 3:**
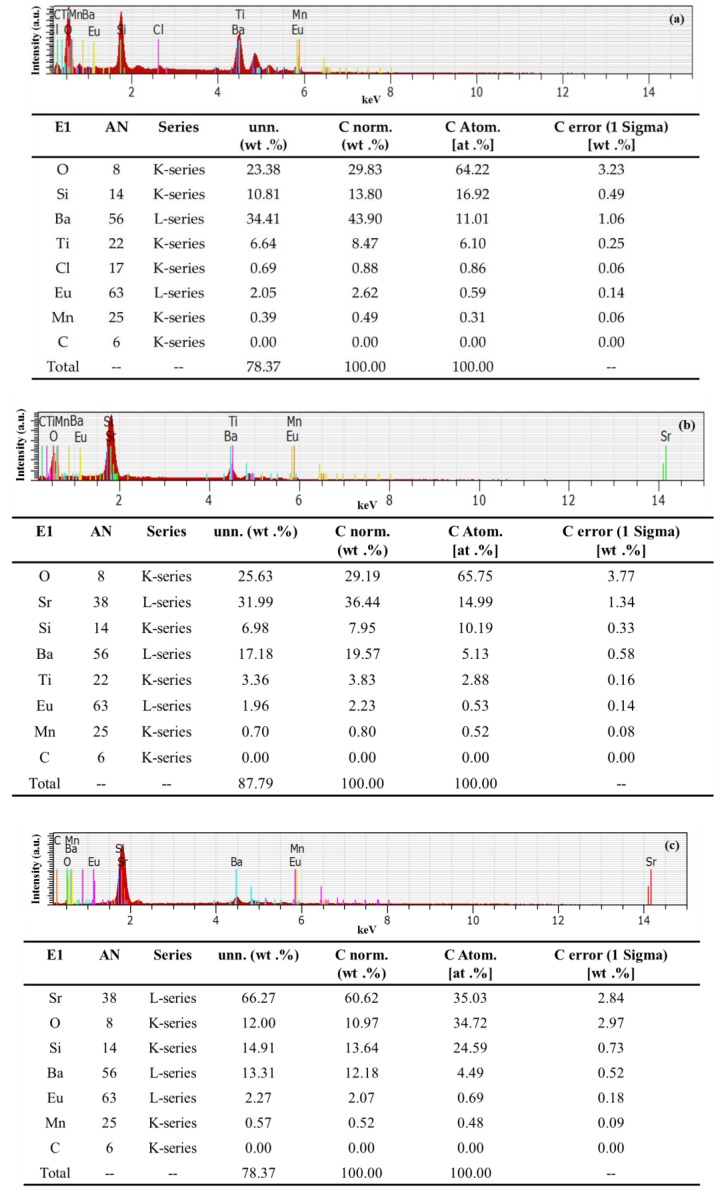
Energy-dispersive X-ray spectroscopy (EDS) of Eu-doped silicate phosphors: (**a**) G2060TM; (**b**) EY4156; and (**c**) O6040.

Energy-dispersive X-ray spectroscopy (EDS) is an analytical technique used for element analysis or chemical characterization. EDS makes use of the X-ray spectrum emitted by a solid sample bombarded by a focused beam of electrons to enable localized chemical analysis. Each element within the sample has a unique atomic structure, which produces a unique set of peaks in its electromagnetic emission spectrum. Figure 3 presents the EDS spectra of Eu-doped silicate phosphors species (a) G2060TM, (b) EY4156, and (c) O6040, with energy peaks corresponding to the various elements in the samples. The Eu-doped phosphors, in this study, were composed mainly of Sr, Ba, Si, Ti, and O with small quantity of Eu and Mn.

**Figure 4:**
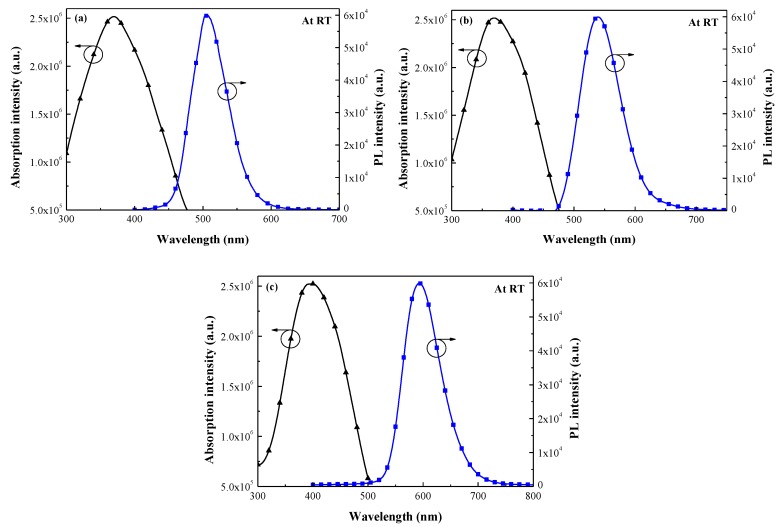
Absorbance and photoluminescence (PL) spectra of 3 wt % Eu-doped phosphors: (**a**) G2060TM; (**b**) EY4156; and (**c**) O6040.

Figure 4 presents the normalized absorption and PL fluorescence emission spectra of Eu-doped phosphors samples: (a) G2060TM, (b) EY4156, and (c) O6040. The absorption and PL emission peaks and the Stokes shift are summarized in Table 2. These results indicate that most of the incident photons at UV–blue wavelengths (300–450 nm) were absorbed by Eu-doped phosphors particles and re-emitted at visible wavelengths (500–650 nm), thereby demonstrating LDS behavior. The Stokes shift of the Eu-doped phosphors was calculated to be >130 nm (from the peak in the absorption spectrum to the peak of the PL spectrum). The Stokes shift of the sample with O6040 was more pronounced than that of the other samples. The downshifted photons (500–650 nm) were re-emitted incident to the cell, which means that they were absorbed closer to the depletion region of the p–n junction. This enhanced the collection of photo-generated charge carriers and suppressed the recombination of charge carriers of short-wavelength (300–450 nm) generated near the surface.

**Figure 5:**
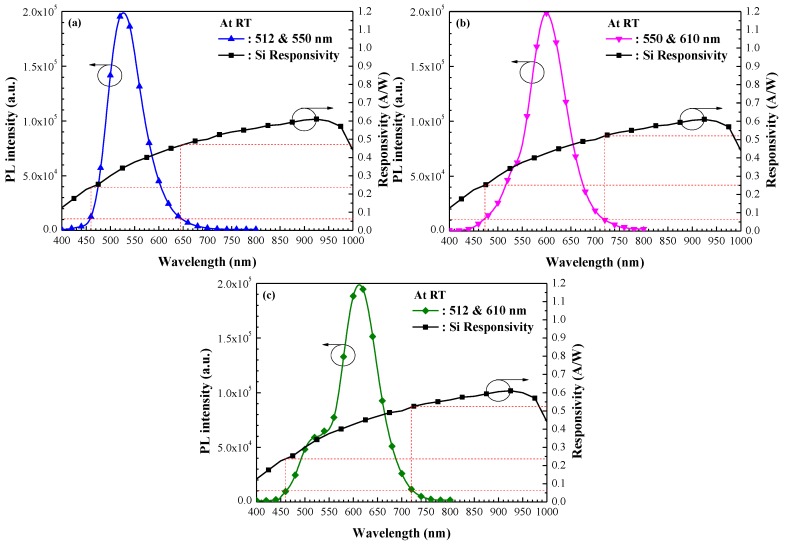
Photo-responsivity spectrum of bare silicon photodiode, and normalized PL emission spectrum of layer with two-species of Eu-doped silicate phosphors with wavelength combinations of (**a**) 512 nm + 550 nm; (**b**) 550 nm + 610 nm; and (**c**) 512 nm + 610 nm.

Figure 5 presents the normalized PL emission spectra of the layer with two-species of Eu-doped silicate phosphors, which resulted in the following emission wavelength combinations: (a) 512 nm + 550 nm, (b) 550 nm + 610 nm, and (c) 512 nm + 610 nm. Table 3 lists the PL emission wavelength range, which is defined as the wavelength range at 5% of the maximum PL intensity of two-species Eu-doped silicate phosphors. The spectral range of photoluminescence emission from the 512/610 nm combination (at 260 nm) exceeded that of the emissions from the 550/610 nm combination (at 248 nm), and that of the 512/550 nm combination (at 185 nm). The PL emission wavelength range of samples with two species of Eu-doped silicate phosphors exceeded those with one species, due to the re-emission of a greater number of photons incidental to the cells. For comparison, Figure 5 also presents the photo-responsivity of a bare silicon photodiode, where a higher value of responsivity indicates higher photo-generated current. As shown in Figure 5, specimens with two types of Eu-doped silicate phosphor layers (512 nm and 610 nm wavelengths) present wider PL emission bands and higher photon emissions at wavelengths where responsivity is high. The broadband LDS performance of cells with two species of Eu-doped silicate phosphors was confirmed using EQE and photovoltaic J–V measurements.

**Figure 6:**
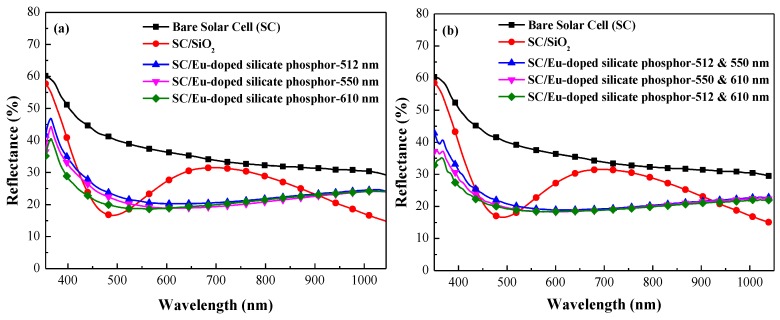
Optical reflectance of bare silicon solar cell, solar cell coated with SiO_2_ layer, and solar cells coated using SiO_2_ layer embedded with (**a**) one species and (**b**) two species of 3 wt % Eu-doped silicate phosphors.

Figure 6 illustrates the reflectivity of a bare silicon solar cell, a cell coated with a SiO_2_ layer, and the silicon solar cells coated with a layer of SiO_2_ mixed with (a) one species and (b) two species of 3 wt % Eu-doped silicate phosphors. The reflectivity of the cells coated with one species was the same as that with two species across the full range of wavelengths. The bare silicon solar cell with a SiO_2_ layer exhibited typical anti-reflectivity with lowest minimum reflectance of 18% at approximately 475 nm due to non-fully destructive interference of the reflected light between the air/SiO_2_ and SiO_2_/Si interfaces. Notice the big difference in the refractive indexes of Silicon (*n* = 4.6) and SiO_2_ (*n* = 1.46) at this wavelength that prevents the reflectance to get a lower minimum value. The reflectance spectrum of the Eu-doped silicate phosphors layer presented a broadband reduction in reflectance from 300 to 1100 nm, far exceeding the performance of the SiO_2_ layer without phosphor particles. The reduction in reflectance at 300–470 nm can be attributed to the absorption of incident light by Eu-doped phosphor particles. We also observed a slight increase in reflectance between 470 and 580 nm, due in part to the re-emission of light associated with LDS effects as well as the less than ideal antireflection characteristics caused by non-uniformity in the spacing of phosphor particles, which reduced the destructive interference of the reflected light between air/SiO_2_ and SiO_2_/Si interfaces. The reduction in reflectance beyond 550 nm can be attributed to the forward scattering of incident light by phosphor particles on the surface. A reduction in the front surface recombination in the cells coated with a SiO_2_ layer or a layer of SiO_2_ mixed with Eu-doped silicate phosphors can be observed by extracting the ideality factor from the J–V measurements (form 1.65 to 1.53) due to the effects of dielectric passivation.

**Figure 7:**
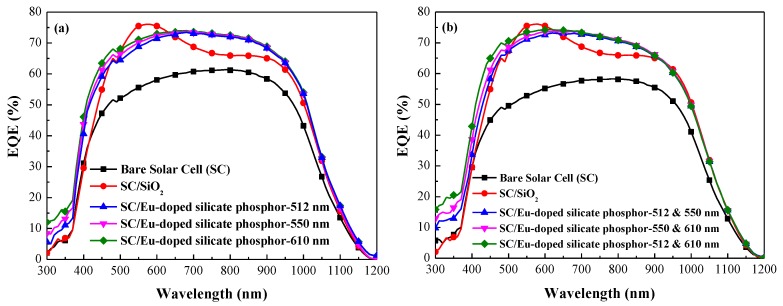
External quantum efficiency (EQE) response of bare silicon solar cell, silicon solar cell with SiO_2_ layer, and silicon solar cells with SiO_2_ layer embedded with (**a**) one species and (**b**) two species of 3 wt % Eu-doped silicate phosphors.

Figure 7 presents the EQE response of the bare silicon solar cell, the silicon solar cell coated with a SiO_2_ layer, and solar cells coated using SiO_2_ layer mixed with (a) one species and (b) two species of 3 wt % Eu-doped silicate phosphors. The EQE responses of the cells with a SiO_2_ layer and the cells with phosphors are in strong agreement with the optical reflectivity results. At wavelengths of 300–470 nm, the EQE values of cells with silicate phosphors were higher than those of cells with only a SiO_2_ layer and the bare cell. This can be attributed to the LDS of Eu-doped phosphor particles. Photons with wavelengths between 300–470 nm are absorbed by phosphor particles and converted into photons within the visible spectrum (480–610 nm), which are far more easily absorbed near the depletion region of the p–n junction. The resulting charge carriers provide far higher collection efficiency, which generates higher photocurrent. We also calculated the average weighted EQE values (EQE_W_) from 320 to 470 nm and from 320 to 1000 nm, as shown in Table 4. The EQE_W_ of the cell with two species of silicate phosphor was higher than that of the cell with one species (due to the effects of broadband LDS), particularly in the cell with two species of Eu-doped silicate phosphors with a wavelength combination of 512 nm and 610 nm. The EQE response results of all cells with silicate phosphors are in agreement with the PL fluorescence emissions.

**Figure 8:**
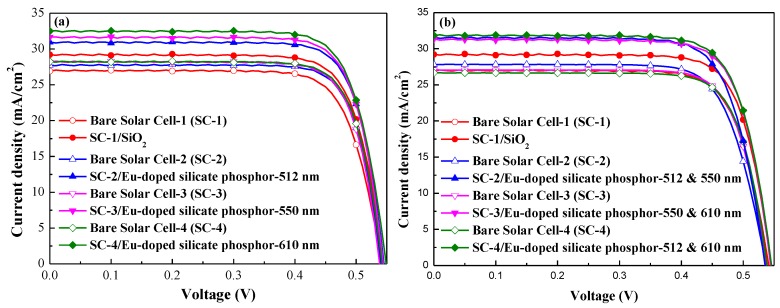
Photovoltaic J–V of bare silicon solar cell, silicon solar cell coated with SiO_2_ layer, and silicon solar cells with SiO_2_ layer mixed with (**a**) one species and (**b**) two species of 3 wt % Eu-doped silicate phosphors.

Figure 8 presents the photovoltaic J–V characteristics of the bare silicon solar cell, the silicon solar cell with SiO_2_ layer, and silicon solar cells using a SiO_2_ layer mixed with (a) one species and (b) two species of 3 wt % Eu-doped silicate phosphors. The photovoltaic performance of the cells is summarized in Table 5a,b. The cell with a SiO_2_ layer outperformed the bare solar cell in terms of short-circuit current density (∆*J*_sc_) by 8.51% (from 26.92 to 29.21 mA/cm^2^), and in terms of conversion efficiency by 10.23% (from 11.49% to 12.28%). The short-circuit current density enhancements (∆*J*_sc_) in cells with one species of Eu-doped silicate phosphors (G2060TM, EY4156, or O6040) were 11.64%, 12.43%, and 15.08%. Similarly, the *J*_sc_ enhancements in cells with two species of Eu-doped silicate phosphors (G2060TM + O6040, EY4156 + O6040, or G2060TM + O6040) were 13.58%, 15.81%, and 19.39%. Compared to the bare reference cell, the ∆*J*_sc_ of 19.39% in the cell with an appropriate combination of two Eu-doped silicate phosphors (G2060TM + O6040) exceeded the 15.08% observed in the cell with only one species of Eu-doped silicate phosphor (O6040) and the 8.51% in the cell with a SiO_2_ layer. The conversion efficiency enhancement (∆*η*) of the cell with a SiO_2_ layer was 10.23% higher than that of the bare reference cell, whereas the cell with one species of phosphor (O6040) was 14.79% higher and the cell with two species (G2060TM + O6040) was 19.35% higher. The ∆*η* value exceeded Δ*J*_sc_ due to an increase in open-circuit voltage (*V*_oc_). This can be attributed to the fact that *η* is proportional to the product of *J*_sc_ and *V*_oc_. Specifically, the cell with two species (G2060TM + O6040) of Eu-doped silicate phosphor layer increased the absolute conversion efficiency by 0.98% over that of the cell with a SiO_2_ layer.

## 4. Conclusions

This study sought to improve the efficiency of crystalline silicon solar cells by coating them with a layer of SiO_2_ mixed with one species or two species of 3 wt % Eu-doped silicate phosphors. We characterized the luminescent down-shifting effects of the phosphor layers according to photoluminescence, optical reflectance, and external quantum efficiency. The photoluminescence emission-band of the samples with two species is wider than that of the samples with one species. The broadest emission-band performance was obtained in samples with two species of phosphors, presenting a combination of emission wavelengths of 512 nm (species of G2060TM) and 610 nm (species of O6040). The conversion efficiency enhancement in the cell coated with two species of Eu-doped phosphors (19.39%) exceeded that of cell coated with one species of phosphor (14.79%) and the cell with a SiO_2_ layer (10.23%).

## Figures and Tables

**Table 1:** Coverage of samples with SiO_2_ layer mixed with one-species or two-species Eu-doped silicate phosphor particles.

Species of Phosphor	Average Coverage (%)
G2060TM	11.97
EY4156	11.87
O6040	12.15
G2060TM + EY4156	11.91
EY4156 + O6040	12.05
G2060TM + O6040	11.75

**Table 2:** Absorption, Photoluminescence (PL) emission peaks, and Stokes shift of 3 wt % Eu-doped phosphors.

Species of Phosphor	Absorption Peak (nm)	PL Emission Peak (nm)	Stokes Shifting (nm)
G2060TM	369	506	137
EY4156	370	539	169
O6040	397	596	199

**Table 3:** PL emission wavelength range of samples combining two-species of Eu-doped silicate phosphors and responsivity of bare silicon photodiode.

Two-Species Combination of Eu-Doped Silicate Phosphors	PL Emission Wavelength Range (nm) @ 5% of Max. Intensity	Responsivity (R; A/W) @ PL Emission Wavelength Range
512 + 550 nm	460–645	0.24–0.47
550 + 610 nm	472–720	0.25–0.52
512 + 610 nm	460–720	0.24–0.53

**Table 4:** Average weighted EQE values (EQE_W_) from 320 to 470 nm and from 320 to 1000 nm.

Silicon Solar Cell	EQE_W_ (%) @ 320–1000 nm	EQE_W_ (%) @ 320–470 nm
Bare Solar Cell (SC)	49.60	32.26
SC/SiO_2_	61.49	37.20
SC/Eu-doped silicate phosphor-512 + 550 nm	62.97	41.45
SC/Eu-doped silicate phosphor-550 + 610 nm	64.22	44.92
SC/Eu-doped silicate phosphor-512 + 610 nm	65.35	48.83

**Table 5:** Photovoltaic performance of bare silicon solar cell, silicon solar cell coated with SiO_2_ layer, and silicon solar cells with SiO_2_ layer mixed with (**a**) one species and (**b**) two species of 3 wt % Eu-doped silicate phosphors.

(**a**)						
**Silicon Solar Cell**	***V*_oc_ (mV)**	***J*_sc_ (mA/cm^2^)**	**Fill Factor (FF) (%)**	***η* (%)**	**Δ*J*_sc_ (%)**	**Δ*η* (%)**
Bare Solar Cell-1 (SC-1)	541.3	26.92	76.45	11.14	8.51	10.23
SC-1/SiO_2_	544.8	29.21	77.22	12.28		
Bare Solar Cell-2 (SC-2)	539.9	27.74	78.79	11.80	11.64	11.69
SC-2/Eu-doped silicate phosphor-512 nm	545.4	30.97	78.07	13.18		
Bare Solar Cell-3 (SC-3)	538.4	28.16	78.54	11.90	12.43	13.03
SC-3/Eu-doped silicate phosphor-550 nm	544.2	31.66	78.08	13.45		
Bare Solar Cell-4 (SC-4)	542.3	28.24	78.21	11.97	15.08	14.79
SC-4/Eu-doped silicate phosphor-610 nm	549.1	32.50	77.01	13.74		
(**b**)						
**Silicon Solar Cell**	***V*_oc_ (mV)**	***J*_sc_ (mA/cm^2^)**	**FF (%)**	***η* (%)**	**Δ*J*_sc_ (%)**	**Δ*η* (%)**
Bare Solar Cell-1 (SC-1)	541.3	26.92	76.45	11.14	8.51	10.23
SC-1/SiO_2_	544.8	29.21	77.22	12.28		
Bare Solar Cell-2 (SC-2)	536.6	27.83	75.0	11.20	13.58	14.02
SC-2/Eu-doped silicate phosphor-512 + 550 nm	535.5	31.61	75.5	12.77		
Bare Solar Cell-3 (SC-3)	537.3	27.08	77.1	11.22	15.81	16.49
SC-3/Eu-doped silicate phosphor-550 + 610 nm	544.3	31.36	76.5	13.07		
Bare Solar Cell-4 (SC-4)	538.8	26.66	77.5	11.11	19.39	19.35
SC-4/Eu-doped silicate phosphor-512 + 610 nm	545.8	31.83	76.4	13.26		
